# Expanding Horizons: Next‐Generation and Interdisciplinary Advances in the Applications of Extracellular Vesicles

**DOI:** 10.1002/jex2.70101

**Published:** 2025-12-14

**Authors:** Esperanza González, Juan Manuel Falcón‐Pérez

**Affiliations:** ^1^ Exosomes Laboratory, Center for Cooperative Research in Biosciences (CIC bioGUNE) Basque Research and Technology Alliance (BRTA) Derio Spain; ^2^ IKERBASQUE Basque Foundation for Science Bilbao Spain; ^3^ Centro de Investigación Biomédica en Red en el Área temática de Enfermedades Hepáticas (CIBEReh) Madrid Spain

**Keywords:** cosmetics, bioremediation, business opportunity, dermatology, ecology, extracellular vesicles, functional food, interkingdom

## Abstract

Extracellular vesicles (EVs) are increasingly recognized as universal mediators of communication in nature across all domains of life and as versatile tools with roles spanning a wide range of industries. Although EVs have been extensively studied in biomedicine, mainly in diagnostic and nanotherapy of cancer and neurodegenerative diseases, their potential applications in other impactful society areas are only beginning to be explored. Microbial EVs contribute significantly to biofilm formation, virulence and the transmission of antibiotic resistance, highlighting their importance in pathogenicity and infection control. In the aesthetic and dermatological sectors, EVs are gaining traction as innovative agents for skin regeneration, anti‐ageing and inflammation modulation, with applications extending to cosmetic dermatology and non‐invasive treatments. Veterinary medicine is also exploring EVs for diagnostics and therapeutic delivery, while in agriculture, they show promise in improving crop resilience, acting as natural biopesticides and supporting plant–microbe interactions. Inter‐species and interkingdom EV communication understanding, potentially help pest control and disease prevention. Moreover, EVs are being investigated as biosensors for environmental pollution and as agents in soil and water remediation. In the food industry, EVs are explored for their functional benefits in promoting gut and systemic health. However, to fully realize their potential, challenges in large‐scale production, quality control and regulatory approval must be addressed. In this article, innovative solutions and potential of EVs across other health issues, environment, agriculture and biotechnology have been revised and discussed.

## 1 Introduction: EVs Everywhere in Nature

**FIGURE 1:**
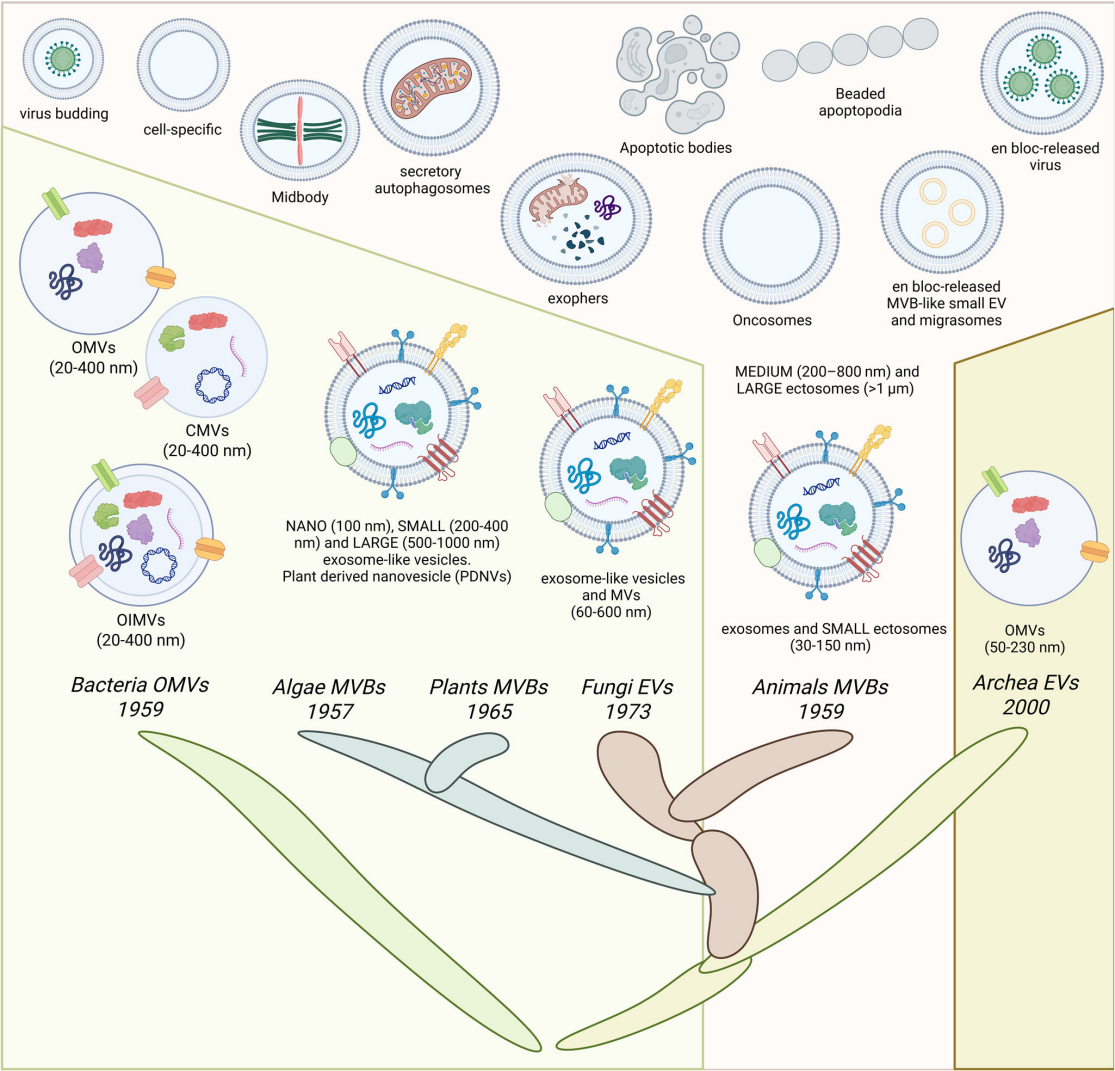
Extracellular vesiscles in EV‐olution. Genealogy tree of the extracellular vesicles described along evolution. Exosomes and small ectosomes (30–150 nm), medium ectosomes (200–800 nm) ‐cell specific and functional ectosomes, secreted midbody remnants and ectosomes containing virus‐ and large ectosomes (>1 µm) ‐exophers, apoptotic bodies, beaded apoptopodia, large oncosomes, en bloc‐released virus clusters, en bloc‐released MVB‐like small EV clusters, migrasomes and secretory autophagosomes‐ in mammals. Nano (∼100 nm), small (200–400 nm) and large (up to 1000 nm) exosome‐like vesicles in plants (P‐EVs). Microvesicles and exosome‐like vesicles (60 to 600 nm) in fungi. Outer membrane vesicles (OMVs), cytoplasmic membrane vesicles (CMVs) and outer inner membrane vesicles (OIMVs) (20–400 nm) in bacteria (b‐EVs). Outer membrane vesicles (OMVs) (50–230 nm) in archaea. Created in https://BioRender.com.

Extracellular vesicles (EVs) are universally released by nearly all cell types across all empires of life, underscoring their ubiquity and fundamental role in life (Figure 1). The earliest observations of EVs date back to the 1950s, beginning with their identification in algae and subsequently in mammalian cells, where intraluminal vesicles within multivesicular bodies (MVBs), the precursors to exosomes, were first noted (Sager and Palade 1957; Sotelo and Porter 1959). Concurrently, outer membrane vesicles (OMVs) were discovered in bacteria (De 1959; Chatterjee et al. 1959) and after, MVBs were identified in higher plants in 1965 (Jensen 1965) and in fungi by 1973 (Takeo et al. 1973). For many years, EVs were thought to serve merely as cellular waste disposal systems (Johnstone et al. 1987), although this perception began to shift in 1996 when EVs were demonstrated that could influence antigen presentation in vivo (Raposo et al. 1996). Their biological relevance was further underscored in 2000 when EVs were identified in archaea, confirming their presence across all domains of life (Prangishvili et al. 2000). All these discoveries laid the foundation for our current understanding of EVs as key players in intercellular communication and various physiological processes.

The term EVs refers to particles that are released from cells, delimited by a lipid bilayer and cannot replicate on their own and transport a wide range of bioactive molecules, including proteins, lipids, metabolites and nucleic acids (Welsh et al. 2024). They play a vital role in intercellular communication, defence and homeostasis by delivering their cargo either through uptake by recipient cells via membrane fusion or endocytosis or by interacting with surface receptors (van Niel et al. 2018).

Mammalian EVs are the most extensively studied and their heterogeneity reflects the diversity of origin and functional state of the releasing cells, together with the distinct biogenetic pathways involved. EVs are broadly classified into two major subtypes: exosomes and ectosomes (Buzas 2023; Jeppesen et al. 2023). Exosomes are formed through the fusion of MVBs or amphisomes (Jeppesen et al. 2019) with the plasma membrane, although other endomembranes are also suggested to be involved, such as endoplasmic reticulum (Barman et al. 2022) and nuclear envelope (Arya et al. 2022). Ectosomes are shed directly from the plasma membrane by budding and blebbing (Mathieu et al. 2019). Exosomes are the smallest subgroup in size (30–150 nm) together with small ectosomes, such as arrestin domain‐containing protein 1‐mediated microvesicles (Wang and Lu 2017). Medium‐sized EVs (200–800 nm) are less abundant and include cell specific and functional ectosomes (Buzas 2023), secreted midbody remnants (Rai et al. 25 2021) and ectosomes containing virus (Van Der Grein et al. 2018). Large EVs (diameter ≥ 1 µm) are the least abundant population of EVs. They comprise an heterogeneus group of vesicles that include exophers (Melentijevic et al. 2017), apoptotic bodies, beaded apoptopodia (Atkin‐Smith et al. 2019), large oncosomes (Di Vizio et al. 2012) and en bloc‐released virus clusters (Kerviel et al. 2021). Other large EVs involve intracellular compartments, such as en bloc‐released MVB‐like small EV clusters (Valcz et al. 2019), migrasomes (Ma et al. 2015) and secretory autophagosomes (van Niel et al. 2022), what suggests that the EV‐ biogenesis and regulatory mechanisms are not yet fully understood. Thus, secretome gains in complexity with the presence of very small non‐EV nanoparticles without a phospholipid bilayer membrane that wrap them, such as exomeres (Zhang et al. 2018) and supermeres (Zhang et al. 2021). Animal EVs are found in all body fluids (blood, saliva, urine, etc.) and play a crucial role in intercellular communication (Jeppesen et al. 2023, Mathieu et al. 2019). The Minimal Information for Studies of Extracellular Vesicles (MISEV) guidelines, initially published in 2014, updated in 2018 and further refined with the release of MISEV2023, provides comprehensive and updated standards for the isolation, characterization and quantification of EVs (Welsh et al. 2024).

EVs have also been identified in a variety of plant species and isolated from multiple organs, including leaves, fruits, seeds, roots/rhizomes and pollen. These plant‐derived EVs or plant derived nanovesicles (PDNVs) are generally categorized into three size‐based groups: nano (∼100 nm), small (200–400 nm) and large (up to 1000 nm). Electron microscopy has confirmed that these vesicles are shed from MVBs, supporting their classification as exosome‐like vesicles. However, there is currently no confirmed evidence of microvesicles production in plants (Pérez‐Bermúdez et al. 2017). PDNVs are found in the apoplast and could be released by mechanical force through the relatively non‐rigid cell walls or by modulation of cell wall architecture as bacteria and yeast do (Casadevall et al. 2009; Brown et al. 2015). PDNVs are released especially under conditions of stress or infection, playing a key role in cell‐to‐cell communication, defence signalling, wound healing and stress responses (Hao et al. 2024).

Descending the evolutionary scale, fungi are also described to release EVs and they display morphological and protein content similarities to mammalian exosomes (Liebana‐Jordan et al. 2021). Evidence suggests fungi produce both MVB‐derived exosomes and membrane‐derived microvesicles between 60 and 600 nm (Rodrigues et al. 2013), akin to the processes observed in animal cells. However, like plant EVs, how fungal EVs traverse the rigid cell wall is still not fully understood. Importantly, fungal EVs are closely associated with virulence and can elicit immune responses when introduced in vivo (Joffe et al. 2016).

Gram‐negative bacteria release OMVs derived from the outer membrane and gram‐positive bacteria produce cytoplasmic membrane vesicles (CMVs). These bacteria EVs (bEVs) rang in size from 20 to 400 nm in diameter (Toyofuku et al. 2019) and contain cell wall components, peptidoglycans, outer membrane proteins, lipopolysaccharides, phospholipids, as well as soluble proteins (periplasmic, cytoplasmic) such as enzymes, nucleic acids (DNA, RNA) and secondary metabolites. Such composition relies on bacterial source and growth conditions, including nutrients availability, temperature, antibiotics and so forth. Functions of bEVs involve delivery of toxins, enzymes and signalling molecules, biofilm formation and structural maintenance, horizontal gene transfer (HGT) and immune evasion (Toyofuku et al. 2023).

EVs have been found to be released by various archaeal species with similarities to a Gram‐negative bacteria's OMVs. Sized in 50–230 nm, archaeal EVs possess a unique envelope composed of ether‐linked lipids that provide enhanced stability in extreme environments such as high temperatures, high salinity and low pH. Additionally, many archaeal EVs are associated with an S‐layer, a paracrystalline proteinaceous structure that influences vesicle stability and may contribute to selective cargo loading. Functionally, archaeal EVs have been shown to prevent DNA thermodenaturation, facilitate HGT and nutrient cycling in extreme environments (Liu et al. 2021).

Because the release of EVs is ubiquitous across all domains of life, their evolutionary advantage is evident. In recent years, EVs have gained considerable attention, particularly for their roles in diagnostics, disease monitoring via liquid biopsy and as vehicles for targeted drug delivery, mainly focused on cancer and neurodegenerative diseases. Currently, over 200 clinical trials are investigating the therapeutic and diagnostic potential of EVs in a wide range of indications, including cancer, COVID‐19, wound healing and degenerative diseases.

**FIGURE 2:**
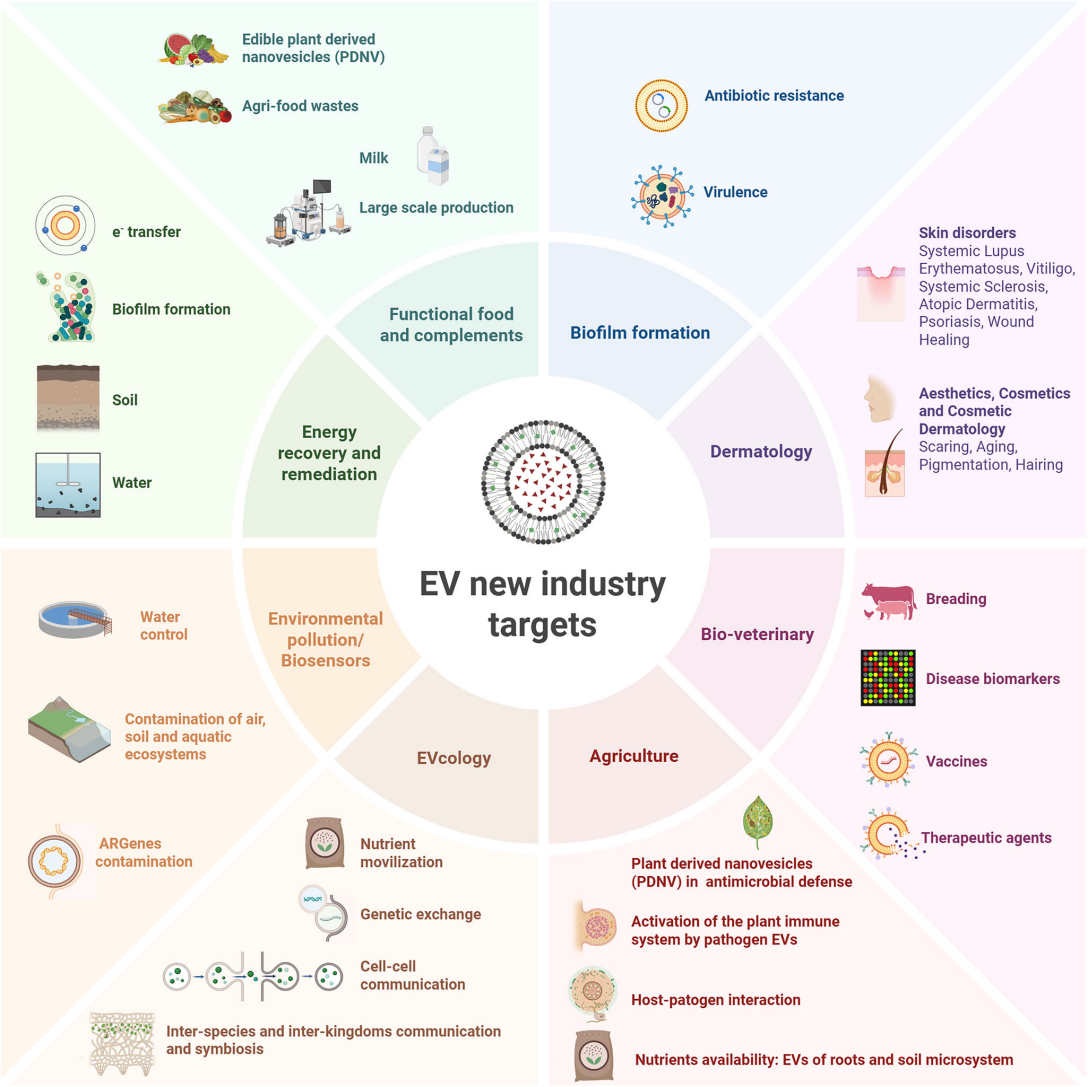
Next generation and innovative interdisciplinary frontiers in extracellular vesicle research. New areas to expand extracellular vesicles (EVs) knowledge include their use dermatology, aesthetic and cosmetics, pathogen persistance by biofilm formation, veterinary applications, agriculture, functional food, ecology, biorremediation and contaminant biosensors, and large scale production, regularization and standarization. Created in https://BioRender.com.

As the field of EVs continues to evolve, new and diverse areas of research and applications are steadily emerging. In this review, we explore the expanding landscape of research and practical applications trends, challenges, and future directions in the rapidly growing field, distinct from classical applications in biomedicine mentioned above. Topics discussed include the role and use of EVs in biofilm formation and pathogenicity, dermatology, aesthetic and cosmetic applications, veterinary and agriculture, ecology, environmental pollution and energy recovery and remediation. Additionally, we explore the promise of food‐derived EVs in modulating gut microbiota and influencing host health (Figure 2).

## 2 Biofilm Formation, Virulence and Antibiotic Resistance of Microbial Pathogens

Microbial pathogens persist in host environments by forming biofilms (Jiang et al. 2024), consisting of organized microbial communities encased in a self‐produced extracellular polymeric substance (EPS) matrix and adhered to biotic or abiotic surfaces. This structure provides microbes with protection from host defences and drug treatment. Given that, biofilms are often resistant to complete eradication (Ciofu et al. 2022).

Bacterial extracellular vesicles (bEVs) play a major role in the establishment of new biofilms by transporting EPS components such as extracellular polysaccharides, proteins and extracellular DNA, helping to promote adherence to surfaces and stabilize the structure (Toyofuku et al. 2023; Schooling and Beveridge 2006). bEVs are also involved in intercellular communication and nutrient transport, key for biofilm grow and maintenance (Turnbull et al. 2016). Moreover, bEVs are responsible of biofilm protection contributing to the physical barrier that limits antibiotic penetration, delivering enzymes like β‐lactamases that degrade antibiotics or sequestrating them. In this line, bEVs participate in HGT transporting and disseminating DNA fragments or plasmids with antibiotic resistance genes. In addition, bEVs contain toxins, virulence factors, and immunomodulatory compounds against the host (Begić and Josić 2020). However, bEVs are known to also obstruct early‐stage biofilm formation. In such microbe structures, bEVs carrying virulence factors and toxins interact, inhibit the growth and kill competing microbial communities leading to the EV‐producer successful competition and nutrient advantage (Turnbull et al. 2016; Jeong et al. 2024). Similarly, biofilm‐derived fungi EVs fulfil a crucial function in biofilm formation and dispersal, including adhesion and grow (Zarnowski et al. 2021; Brandt et al. 2024). Furthermore, fungi EVs are involved in biofilm drug tolerance, as well as in community coordination as *wild‐type* strains can balance mutant phenotypic defects via EV‐exchange of genetic material (Zarnowski et al. 2021).

As bacteria and fungi EVs module biofilm establishment, integrity and success, they emerge as target for developing novel antimicrobial therapies and diagnostics. Understanding EVs in biofilms has important applications in diagnostics as biomarkers for biofilm‐related infections, in therapeutics as targets to disrupt biofilms and in drug delivery, using engineered EVs to penetrate biofilms, which is an important challenge in medical devices and odontology.

## 3 Dermatology

Following the era of plasma‐based therapies in dermatology and in aesthetic and cosmetic industry, there is now growing attention on EVs from those plasma (Anitua et al. 2023) and from other sources given their rich composition of proteins, lipids and various molecules that alleviate inflammation and foster tissue repair, hydration and skin protection from environmental damage. All this make EVs an opportunity in regenerative medicine and skin rejuvenation (Kee et al. 2022; Thakur et al. 2023; Yousefian et al. 2024). Due to their therapeutic features and capability of penetrating the skin barrier to enhance cosmetic ingredient delivery, utilization of EVs in creams, serums, masks and so forth, for topic use is currently in development (Yousefian et al. 2024).

### 3.1 Applications in Skin Disorders

EVs have emerged as key regulators and alleviation of the pathogenesis of numerous dermatological conditions, including Systemic Lupus Erythematosus (SLE), Vitiligo, Systemic Sclerosis (SSc), atopic dermatitis (AD, eczema), psoriasis and wound healing.

#### 3.1.1 Systemic Lupus Erythematosus (SLE)

SLE is a chronic autoimmune disease that can affect multiple organs and systems in the body, leading to a wide range of symptoms and complications, including inflammation and immune dysregulation (Dai et al. 2025). EVs have emerged as key players in the pathogenesis and progression of SLE (Cojocaru et al. 2011). In patients with SLE, EVs from immune and endothelial cells transport antigens, autoantibodies and inflammatory molecules, contributing to tissue damage (Lee et al. 2016). Given their immunomodulatory roles, EVs represent both a pathogenic factor and a promising target for therapeutic intervention in SLE.

Mesenchymal stem cell‐derived EVs (MSC‐EVs) have shown promise in SLE due to their anti‐inflammatory and immunosuppressive properties. These vesicles can deliver bioactive molecules such as microRNAs, cytokines and anti‐inflammatory proteins that modulate T‐cell activation, reduce autoantibody production and restore regulatory immune responses (Lv et al. 2025; Yang et al. 2021; Phinney and Pittenger 2017). EVs could be also loaded with anti‐inflammatory or immunosuppressive molecules to reduce the production of antibodies that can attack the body's tissues patients (Liu et al. 2023). Additionally, their low immunogenicity and ability to cross biological barriers make them suitable candidates for repeated administration (Wu et al. 2025).

#### 3.1.2 Vitiligo

Vitiligo is an autoimmune skin condition that causes the loss of skin pigment, resulting in white patches on the skin (Picardo et al. 2015; Picardo 2023). EVs from melanocytes, keratinocytes and immune cells may influence melanocyte destruction through affecting gene expression related to melanin synthesis and modulating immune responses. Certainly, they may carry antioxidant molecules that help mitigate oxidative stress (Wong et al. 2020), protect the skin from UV‐induced damage and contribute to the repair of damaged skin cells, which make EVs promise as novel therapeutic tools for vitiligo treatment (Perez‐Bootello et al. 2023).

MSCs regulate cytokine secretion and modulate the balance of T‐cell subsets, thereby mitigating immune‐mediated destruction of melanocytes (Zhu et al. 2020). EVs released from MSCs mediate immunomodulatory effects (Chang et al. 2021). These properties position MSCs and their EVs as a promising future therapeutic option for vitiligo.

#### 3.1.3 Systemic Sclerosis (SSc)

SSc is a chronic autoimmune disease marked by fibrosis of the skin and internal organs, vascular abnormalities and immune dysregulation (Son and Moon 2025). Specifically, skin fibrosis in SSc primarily results from the overproduction of extracellular matrix by activated dermal fibroblasts (Jimenez et al. 2025). EVs seems to have a role in all these three main aspects, such endothelial disfunction and fibrosis (Colletti et al. 2019). EVs from SSc fibroblasts stimulated the increasing levels of type I collagen in normal fibroblasts, which contribute to skin regeneration (Nakamura et al. 2016).

Although current therapies can help manage the symptoms of SSc, they do not offer a cure. Interestingly, emerging research on MSC‐EVs presents a promising breakthrough, as these vesicles exhibit anti‐fibrotic, pro‐angiogenic and immunomodulatory properties that may alleviate fibrosis and support vascular regeneration (Wang et al. 2024). Additionally, platelet‐rich plasma (PRP) has also shown potential therapeutic effects in SSc (Nilforoushzadeh et al. 2025), making it worthwhile to investigate the specific role of PRP‐derived extracellular vesicles (PRP‐EVs) in mediating these effects (Gupta et al. 2025).

#### 3.1.4 Atopic Dermatitis (AD)

AD, widely known as eczema, is a chronic inflammatory skin condition marked by symptoms including intense itching (pruritus), dryness (xerosis) and skin inflammation (Narla and Silverberg 2025). EVs released by resident opportunistic microbes in damaged skin contribute to AD condition by stimulating dermal fibroblasts to produce pro‐inflammatory mediators and destroying keratinocytes. This, together with EV‐antibiotic resistance HGT, contributes to the persistence and severity of chronic infections associated with AD. All cooperatively compromises epidermal integrity that allows, in turn, deeper penetration of pathogens (Harvey‐Seutcheu et al. 2024).

Topical application of adipose‐derived stem cell extracellular vesicles (ASCs‐EVs) has demonstrated promising effects in mitigating the symptoms of AD, reducing erythema and inflammation, enhancing skin barrier integrity and promoting angiogenesis (Cho et al. 2018). Mesenchymal stromal cells derived EVs (MSSCs‐EVs) are also involved in immunomodulation, skin regeneration, barrier repair and microbiome regulation (Al‐Masawa et al. 2025).

EVs from beneficial microbes and marine organisms have shown promising therapeutic effects in AD models. In vitro and oral administration of *Lactobacillus plantarum*‐derived EVs (LpEVs) alleviated *Staphylococcus aureus* EV‐induced skin inflammation, reduced epidermal thickening and lowered IL‐4 levels (Kim et al. 2018). Similarly, EVs from *Limosilactobacillus fermentum* SLAM216 (LF216) promoted wound healing in keratinocyte cultures and suppressed production of pro‐inflammatory cytokines. These EVs also modulated serotonin pathways via gut microbiota alterations, contributing to reduced scratching behaviour and improved depression‐related symptoms in AD models (Choi et al. 2025). Additionally, EVs isolated from the mucus of *Pinctada martensii*, a pearl‐producing mollusk, significantly decreased reactive oxygen species (ROS), lysosomal damage and lactate dehydrogenase (LDH) release in keratinocytes, demonstrating strong anti‐inflammatory effects. In an AD mouse model, these EVs enhanced skin healing, reduced inflammation and thickness and improved collagen restoration (Wu et al. 2024).

#### 3.1.5 Psoriasis

Psoriasis is a chronic immune skin disorder characterized by inflammation, scarring and rapid keratinocyte proliferation. Beyond skin involvement, it can affect joints and other organs. Although not life‐threatening, it often causes significant cosmetic and psychological distress, reducing patients' quality of life (Griffiths et al. 2021). EVs have been found to transmit psoriasis‐related inflammatory signals between keratinocytes and immune cells and promote inflammation and disease progression. However, reduced EV production by keratinocytes as putative therapy has yielded conflicting results across different studies (Jiang et al. 2024).

EVs from healthy adipose‐derived MSCs (ASCs‐EVs) significantly reduced erythema, induration, and skin thickness in psoriasis patients (Mohseni Meybodi et al. 2024). Topical administration of MSC EVs show both anti‐inflammatory and immune‐modulating activity (Lai et al. 2023). IFN‐γ‐stimulated MSC‐derived EVs and UCB‐derived mononuclear cell EVs reduced the inflammatory molecular phenotype (Zhang et al. 2022; Rodrigues et al. 2021). On the other hand, PD‐L1–enriched EVs have demonstrated effectiveness as targeted drug delivery vehicles to PD‐1–expressing keratinocytes and immune cells in psoriasis. This strategy significantly reduced psoriasis symptoms in mice by decreasing epidermal thickness, reducing plaque formation and suppressing excessive inflammatory responses (Jia et al. 2023; Dehghani 2024). Collectively, these findings underscore the potential of EVs as targeted and effective delivery systems for anti‐psoriatic therapies.

#### 3.1.6 Wound Healing

Wound healing is a multifaceted biological process that restores damaged tissue through a coordinated sequence of phases: haemostasis, inflammation, proliferation and remodelling (Dehghani 2024). The role of EVs in wound repair is reflected in accelerating wound haemostasis, regulating the anti‐inflammatory polarity of macrophages, stimulating the proliferation and migration of the vascular endothelial cells and fibroblasts, regulating the cytokine ratio and remodelling the ECM (Eerdekens et al. 2025).

EVs from various sources have shown promising potential in wound healing repair, including those derived from MSCs (Eerdekens et al. 2025; Ding et al. 2023). One study revealed that EVs from bone marrow‐derived stem cells (BMSCs‐EVs), umbilical cord stromal cells (UCSCs‐EVs) and dermal‐derived stem cells (DSCs‐EVs) demonstrated superior wound healing effects, surpassing those of adipose tissue derived stromal cells (ASC‐EVs) and dental pulp stem cells (DPSC‐EVs), as evidenced by smaller wound areas and accelerated closure rates (Li et al. 2023). EVs from hair follicle‐derived mesenchymal stromal cells (HF‐MSCs) were demonstrated to exhibit comparable therapeutic potential for chronic wound healing to that of ASC‐EVs (Las Heras et al. 2022). Macrophage derived EVs, endothelial cells derived EVs (E‐EVs), platelet derived EVs (P‐EVs), erythrocyte derived EVs (RBC‐EVs), plant derived EVs or nanovesicles (PDNVs) and Engineered EVs are also attractive option for would healing alleviation (Sarcinella et al. 2023). These findings underscore the therapeutic potential of EV‐based approaches in advancing wound care chronic or severe wounds and tissue regeneration.

### 3.2 Aesthetic, Cosmetic and Cosmetic Dermatology Applications

EVs offer promising applications in non‐invasive skin rejuvenation and repair due to their ability to regulate cellular communication and tissue regeneration in scaring, ageing, pigmentation disorders and hair renewal (Kee et al. 2022).

#### 3.2.1 Scarring

Hypertrophic scars and keloids are benign fibroproliferative conditions that develop after skin injury, marked by excessive fibroblast activity and abnormal extracellular matrix (ECM) accumulation (Andrews et al. 2016).

EVs derived from adipose tissue derived mesenchymal stem cells (ASCs‐EVs) inhibit the migration and proliferation of hypertrophic scar derived fibroblasts (HSFs) in vitro and reduce collagen deposition in a full‐thickness mouse wound model, thereby attenuating fibrosis (Li et al. 2021). Human amniotic epithelial cell EVs also suppress hypertrophic scar formation (Zhao et al. 2017). Apart from being implicated in wound healing closure, EVs can aid in the treatment of scars as reduce fibrosis and modulate extracellular matrix remodelling beyond modulating cell activity, inflammation and angiogenesis (Zhong et al. 2023). A growing body of preclinical research, along with ongoing clinical trials, supports the safety, efficacy and therapeutic potential of EVs‐based treatments for scar reduction in humans.

#### 3.2.2 Aging

Skin aging, influenced by genetic and extrinsic environmental factors, leads to visible signs such as wrinkles, pigmentation and sagging. Skin aging involves weakened dermal structure due to reduced fibroblast activity and decreased production of collagen, elastin and fibronectin. It also impairs epidermal renewal, causing thinning and abnormal keratinocyte behaviour. Aging disrupts collagen homeostasis, with decreased collagen I, increased collagen III, and elevated MMPs, leading to ECM degradation. These changes contribute to collagen and elastic fibre disorganization, resulting in skin laxity and wrinkle formation. Particularly, photoaged skin shows irregular thickness, pigmentation changes, collagen loss and elastic fibre damage (Chaudhary et al. 2020).

EVs have shown significant potential in anti‐aging applications due to their ability to modulate cellular behaviour and promote tissue regeneration by promoting collagen synthesis, inhibiting matrix degradation and reducing oxidative stress, what would support anti‐ageing therapies (Manni et al. 2023). Human iPSC‐EVs ameliorated aged human dermal fibroblasts (HDFs) in vitro in terms of proliferation, viability and senescence‐related gene expression (Oh et al. 2018). When applied to senescent MSCs, iPSC‐EVs reduce intracellular ROS levels and alleviate their aging, at least in part, by peroxiredoxins (Liu et al. 2019). Incubation of old MSCs with young MSC‐derived EVs decreased aging markers and increased pluripotency markers (Fafián‐Labora et al. 2020). ASCs‐EVs alleviate senescence phenotype in HDFs (Guo et al. 2022, Liang et al. 2020).

Beyond skin alterations, aging induces more profound systemic changes. Old mice treated with small extracellular vesicles (sEVs) derived from adipose mesenchymal stem cells (ASCs) of young animals presented proregenerative properties and reduced oxidative stress, inflammation and senescence markers in muscle and kidney, as well as epigenetic and metabolic age. This was also translated in beneficial functional outcomes in term of motor coordination, grip strength, fatigue resistance, fur regeneration, renal function and frailty (Sanz‐Ros et al. 2022).

In addition to cultured cells, other EV sources have been explored. Milk‐derived EVs possess anti‐aging properties on keratinocytes and fibroblasts, resulting in wrinkle reduction and skin hydration (Lu et al. 2024). Bovine colostrum EVs boost fibroblast proliferation while senescence phenotype (Han et al. 2022). Mushroom‐derived EVs contain inhibit photoageing and provide UV protection by modulating gene expression in skin cells (Han et al. 2022). Apple‐derived nanovesicles exhibit anti‐inflammatory and restructuring effects in skin (Trentini et al. 2022).

EVs provide a cell‐free approach to combat skin ageing. As research progresses, these vesicle‐based therapies hold promise for targeted and regenerative anti‐ageing solutions.

#### 3.2.3 Hyperpigmentation Disorders

Hyperpigmentation is a prevalent skin disorder characterized by the dark spots and patches in the skin (Thawabteh et al. 2023). This occurs due to issues in melanin synthesis, melanosome transportation to adjacent keratinocytes and/or melanosome degradation (Zolghadri et al. 2023).

EVs secreted by keratinocytes are demonstrated to modulate pigmentation in melanocytes (Cicero et al. 2015). From a therapeutic perspective, EVs derived from ASCs effectively reduced intracellular melanin levels in melanocytes and formulated for topical administration, successfully reduced hyperpigmentation (Cho et al. 2020). Alternatively, EVs from the Korean seaweeds *Sargassum fusiform* and *Codium fragile* and milk‐derived EVs reduce melanin synthesis and downregulate melanogenesis‐related genes such as *Tyrosinase* (Jang et al. 2021; Bae and Kim 2021).

#### 3.2.4 Hair

Alopecia is the medical term for hair loss from the scalp or other parts of the body. It can occur in various forms, with differing causes and severity. Dermal papilla cells (DPCs), located at the base of hair follicles, secrete signals and growth factors that regulate the proliferation and differentiation of nearby epithelial cells. This reciprocal communication is essential for hair follicle development and the regulation of the hair growth cycle (Driskell et al. 2011).

EVs secreted by DPCs enhance the proliferation of dermal papilla and outer root sheath cells and increase hair shaft elongation in cultured human hair follicles (Kwack et al. 2019). ASCs‐EVs improve hair follicle regeneration in vivo (Wu et al. 2021). Similarly, MSCs‐EVs stimulate the proliferation and migration of DPCs and facilitate hair growth in vivo (Liu et al. 2024). Likewise, EVs derived from other cell types, such as fibroblasts and macrophages, have also been shown to influence hair growth (Li et al. 2021). While FDA‐approved treatments for moderate hair loss are commonly used, EVs‐based therapies present a promising, targeted alternative for promoting hair regrowth.

## 4 Veterinary

While still emerging, research in EVs shows promise veterinary medicine, especially in regenerative therapies and reproductive health (Moccia et al. 2022). Key applications include musculoskeletal injuries in horses and dogs, wound healing, joint diseases like osteoarthritis (in horses and dogs), mastitis (in cows) and reproductive health in livestock (Xiong et al. 2024). Other areas of interest include neurodegenerative diseases, liver disease and diabetes (Koprivec and Majdič 2024).

Perhaps the main area of research in animals is reproduction. EVs facilitate intercellular communication during reproduction across a range of animal species, playing critical roles in reproductive processes, including sperm maturation and viability, oocyte maturation, conceptus implantation and the establishment of a successful pregnancy. Emerging evidence also links EVs to reproductive pathologies such as pregnancy loss, polycystic ovary syndrome and endometriosis (Godakumara et al. 2022; Mazzarella et al. 2025; Luis‐Calero et al. 2024; Siles‐Lucas et al. 2017). In livestock, EVs present in seminal plasma and follicular fluid contribute to fertility, embryo development and sperm‐egg communication (Martínez‐Díaz et al. 2024). In hens, uterine fluid‐derived EVs have been suggested to support sperm survival (Riou et al. 2020). In felines, follicular fluid‐derived EVs have been shown to enhance oocyte cryopreservation efficiency (de Almeida Monteiro Melo Ferraz et al. 2020). Notably, oviductal EVs have been demonstrated beneficial effects on post‐thaw quality of cryopreserved sperm in species like red wolves and cheetahs, as well as on the motility of red wolf sperm (de Almeida Monteiro Melo Ferraz et al. 2020). In boars, the RNA cargo of seminal plasma EVs has been linked to immune‐related pathways (Bai et al. 2018).

EVs have been isolated from various reproductive tissues of animals and play an important role as biomarkers of fertility in the basis on placental quality, embryo quality and early abortion (Llobat 2021). Indeed, EV‐associated miRNAs present in uterine fluid and serum have been identified as promising biomarkers for pregnancy detection and monitoring in swine (Hua et al. 2021; Zhou et al. 2020) and goats (Zhao et al. 2021). In cattle, EV‐derived miRNAs may also serve as indicators for identifying and selecting developmentally competent blastocysts and embryos (Mellisho et al. 2017; Melo‐Baez et al. 2020). As in humans, the miRNA cargo of EVs has also been investigated in animals for its potential use in liquid biopsy applications. In dogs, EV‐derived miRNAs have been explored as diagnostic and prognostic biomarkers for cancers such as glioblastoma (Narita et al. 2020) and lymphoma (Garnica et al. 2020), and have also proven effective for monitoring cardiotoxicity when undergoing long‐term doxorubicin treatment (Beaumier et al. 2020). Beyond cancer, EV‐associated miRNAs have been studied as biomarkers for other diseases. Urinary EVs have been used to assess kidney dysfunction in dogs (Pomatto et al. 2017) and transferrin receptor (TfR) on serum EV membranes as a biomarker for anaemia in horses (Rout et al. 2015). Milk‐derived EVs are emerging as valuable sources of biomarkers in veterinary medicine. These naturally occurring, membrane‐bound vesicles are rich in proteins, lipids and nucleic acids, including microRNAs and other small RNAs that can provide insights into the physiological status and health of dairy animals. As they reflect the conditions of the mammary gland and systemic health, milk‐derived EVs have the potential to serve as non‐invasive biomarkers for monitoring mastitis, metabolic diseases and general immune status in livestock. Their accessibility and stability in milk make them particularly suitable for veterinary diagnostics and health management in the dairy industry (Rahman and Inoshima 2025).

For therapy in animals, mainly MSCs from adipose tissue (ASCs) and bone marrow (BMSCs) are used as sources of EVs due to their anti‐inflammatory and regenerative properties. However, other sources such as synovial fluid, endometrium, gingiva and milk have been considered (Lanci et al. 2024). Canine BMSC‐derived EVs have demonstrated enhanced wound‐healing efficacy compared to their source cells (El‐Tookhy et al. 2017), while ASC‐derived EVs exhibited notable anti‐inflammatory activity (Oontawee et al. 2025) and therapeutic potential in canine renal ischemia–reperfusion injury (Liu et al. 2023). In equine models, BMSC‐derived EVs have been suggested to possess therapeutic potential for chondrocyte repair (Arévalo‐Turrubiarte et al. 2022) and ASC‐derived EVs have shown beneficial effects in the treatment of suspensory ligament injury (Kornicka‐Garbowska et al. 2019). In veterinary medicine, EVs of blood and immune origin have attracted increasing therapeutic potential. EVs from human PRP enhanced proliferation and matrix deposition while reducing apoptosis in canine tenocytes (Qi et al. 2020), and EVs from canine M1‐polarized macrophages displayed antitumor activity against melanoma and osteosarcoma cells, suggesting their value for EV‐based cancer therapy (Lee et al. 2021). Other EV‐based approaches and advanced EV technologies are also being explored in mice models; however, most of these studies are primarily focused on applications in human nanomedicine.

EVs have emerged as promising tools for vaccine development in veterinary medicine. Antigen‐loaded EVs released by dendritic cells have demonstrated protective effects against avian coccidiosis (Del Cacho et al. 2011). Moreover, EVs originating from pathogens can elicit strong cellular and humoral immune responses (Schorey et al. 2015) and serve as effective antigen delivery vehicles (Micoli and MacLennan 2020). In particular, bEVs carry pathogen‐associated molecular patterns (PAMPs) capable of triggering immune responses against infectious agents (Gnopo et al. 2017). Current investigations are further exploring EV‐based vaccines strategies targeting major animal diseases, including porcine reproductive and respiratory syndrome virus (PRRSV), avian influenza, foot‐and‐mouth disease and other significant infectious diseases (Moccia et al. 2022).

Beyond livestock, EVs from animal species with distinctive and complex adaptive immune systems are increasingly being investigated. In marine fishes, plasma‐derived EVs from cartilaginous species such as the nurse shark (Criscitiello et al. 2019) and mucose‐derived EVs from teleost like farmed cod contain proteins involved in natural immunity against aquatic pathogens (Magnadóttir et al. 2019). In marine mammals, serum‐derived EVs from seals and cetaceans transport critical miRNAs and proteins associated with immunity and metabolism (Magnadóttir et al. 2020; Magnadóttir et al. 2020). Similarly, EVs from seabird plasma carry proteins related to immune and metabolic pathways (Phillips et al. 2020). In reptiles, plasma EVs from crocodiles also carry protein cargo revealing functions beyond immunity (Criscitiello et al. 2020). Overall, these findings indicate that EVs could serve as novel biomarkers of immunological and physiological status across the animal kingdom.

## 5 Agriculture

Plant EVs were really discovered in clumps of carrot suspension cells in 1967. While non‐dividing cells inside clumps were vacuolated, peripheral shown a meristematic phenotype containing MVBs that fused with plasmalemma and released their intraluminal vesicles (ILV) to the cell wall space (Halperin and Jensen 1967). However, such exosome‐like EVs type was firstly isolated from the apoplastic fluid of sunflower (Regente et al. 2009). Since then, EVs have been isolated from diverse plant species, including *Arabidopsis* and named plant‐derived nanovesicles (PDNVs) (Ambrosone et al. 2023).

PDNVs from *Arabidopsis* were found to contain several well‐studied pattern recognition receptors (PRRs) (Yu et al. 2017) and related proteins presumably able to detect PAMPs and activate defence mechanisms (Rutter and Innes 2017). Indeed, EVs from sunflower plants triggered phytopathogenic fungus growth suppression and cell death (Regente et al. 2017). Moreover, EVs released by plants have been demonstrated to contain RNAs and proteins that target pathogens (fungi, bacteria, viruses) (Cai et al. 2018; De Palma et al. 2020; Wang et al. 2024; Davila and He 2025).

In fungal infections, PDNVs‐associated small RNAs (sRNAs) and messenger RNAs (mRNAs) enhance plant disease resistance by silencing fungal virulence genes and activating plant defence responses (Cai et al. 2018). EV‐annexins ANN1 and ANN2 have been described that play a role in stabilizing such RNA molecules, while RNA‐binding proteins (RBPs) are involved in selecting sRNAs for their EV‐ enrichment (He et al. 2021). Other PDNVs‐associated mRNAs can successfully be translated within pathogens into proteins that restrict infection in host plants (Wang et al. 2024). RNA species as tiny RNAs (tyRNAs, 10–17 nt), long non‐coding RNAs (lncRNAs) and circular RNAs (circRNAs) remain to be studied in‐depth (Zhang et al. 2024). PDNVs‐in bacterial infection are less studied, although it is known that both infection and treatment with the defence hormone salicylic acid increase EVs release to the apoplast in *Arabidopsis* (Rutter and Innes 2017).

Likewise, fungal phytopathogen EVs can be taken up by plant cells and contain sRNAs (He et al. 2023), mRNA (Kwon et al. 2021) and metabolites (Garcia‐Ceron et al. 2023) associated with plant immunogenicity. Thus, they induce phytotoxic responses (Bleackley et al. 2019), activate innate immune responses (Zhu et al. 2023) and suppress plant immunity‐related genes (Weiberg et al. 2013), increasing disease susceptibility. In addition, plant pathogenic bacteria outer microvesicles (OMVs) contain not only PAMPs (Bahar et al. 2016) but also numerous infection related proteins (Solé et al. 2015), and they can integrate into plant plasma membrane (Tran et al. 2022) for cargo releasing. In agreement with this, OMVs have been found to activate a broad‐spectrum immune response in plants against bacteria (Bahar et al. 2016, Chalupowicz et al. 2023). Interestingly, the release of OMVs *by Xylella fastidiosa* inhibits bacterial attachment to plant cell walls, as a strategy for spreading and systemic infection (Ionescu et al. 2014).

EVs from pathogens trigger plant immune priming, enhancing resistance to future threats (Ghaffari et al. 2025). Furthermore, PDNVs could be internalized by other plant cells, as demonstrated in tobacco (Kocholata et al. 2022), suggesting a role in pathogen recognition and transmission of immune signals or even of infection to neighboured cells. Indeed, EVs from plants infected with Turnip mosaic virus (TuMV) carry the viral RNA and proteins implied in virus replication and infection (Movahed et al. 2019).

EVs also participate in microbe‐plant communication in soil and root‐associated environments (e.g., rhizobia, mycorrhizae), facilitating symbiosis (Janda and Robatzek 2022). EVs are consistently produced throughout the entire symbiotic lifecycle of both plants and symbiotic fungi and accumulate at the plant–fungus interface during the interaction (Roth et al. 2019; Holland and Roth 2023). In plant‐bacterial interaction, OMVs modulate the expression of nodulation genes related to early symbiosis (Li et al. 2022). Interestingly, plant tetraspanins exhibit transcriptional changes during mycorrhizal association (Jimenez‐Jimenez et al. 2019; Parra‐Aguilar et al. 2023). In addition to this, EVs mediate communication during abiotic stress (e.g., drought, salinity) and carry stress‐response molecules like heat shock proteins and miRNAs that help plants adapt to environmental challenges (Rutter and Innes 2017).

EVs in agriculture are a promising frontier for sustainable farming, offering natural tools for plant protection (Liu et al. 2021), growth stimulation and stress resilience, with potential to replace or reduce chemical inputs (Zhou et al. 2022). Host‐Induced Gene Silencing (HIGS) is a genetic technique in plant biotechnology where the host produces double‐stranded RNA (dsRNA) to silence essential genes in invading pests or pathogens, reducing their ability to infect or survive (Karimi and Innes 2022). EVs are being studied in crop protection as natural pesticide alternatives, delivering RNAs to pests or pathogens without introducing crop genetic modifications (Jang et al. 2024), enabling targeted delivery with minimal environmental impact. Their use in spray‐induced gene silencing (SIGS) offers a promising RNA interference (RNAi)–based approach against eukaryotic plant pathogens (Zhang et al. 2024; Singla‐Rastogi et al. 2019). Other area of research is exploring plant‐derived EVs as natural nanocarriers for delivering pesticides and fertilizers (Zhang et al. 2024). Additionally, EVs could be used to monitor fruit and vegetable health and extend shelf life via EV‐based coatings or treatments that reduce spoilage or microbial contamination. Likewise, they also hold promise as biostimulants, promoting growth and health using EVs from beneficial microbes or plant extracts (Li et al. 2024).

## 6 EVcology

EVs are key messengers that mediate communication not only between cells within an organism but also across species and kingdoms. By delivering bioactive molecules such as proteins, lipids, and nucleic acids, EVs influence the behaviour of recipient cells and form a complex communication network at intercellular, interspecies and interkingdom levels. Inter‐species/kingdoms cell–cell communication including nutrient and genetic exchange is possible thorough EV shuttling (Li et al. 2024; Cai et al. 2023).

EV ecology has been mainly studied in bacteria that colonize soil, aquatic ecosystems and air in term of EV distribution and material transference (Ahmed and McKay; Biller et al. 2023; Yun et al. 2025). bEVs play key roles in both bacterial and host interactions, aid bacterial adaptation to environmental conditions and perform diverse functions such as cell‐to‐cell communication, waste secretion, gene exchange, defence, growth support and electron transfer (Ahmed and McKay). Interestingly, marine bacteria secrete EVs that persist in seawater containing strain‐specific DNA transposons that facilitate HGT of metabolic and phage resistance genes between strains (Hackl et al. 2023). Algal species release sRNA‐containing vesicles during viral infection that affect the dynamics of surrounding microbial populations (Schatz et al. 2021).

EV ecosystems imply biological material transfer and sRNA trafficking that induces gene silencing *in trans* between plants and fungi, bacteria, oomycetes and parasites as referred above (Cai et al. 2023). Interestingly, EVs from nematodes, bacteria and yeast traffic EV biological material into their mammalian hosts, but also EV mammalian hosts traffic sRNAs to interacting organisms (Cai et al. 2023). Given the current understanding of ecosystem complexity, the secretion of EVs into the environment by various organisms offers a new perspective on interspecies and interkingdom communication.

## 7 Biosensors of Environmental Pollution

Pollutants, both man‐made and natural, can cause or worsen cardiovascular, respiratory conditions or other kind of illnesses. Oxidative stress and chronic inflammation are key mechanisms behind their toxicity. Remarkably, pollutants play a significant role in modulating EVs dynamics, influencing their biogenesis, release and cargo composition.

Air pollution is strongly linked to increased daily mortality from cardiopulmonary diseases due to human exposure to harmful gases and particles. EVs released by airway lung cells, epithelial cells and immune cells respond to air pollutants (e.g., particulate matter, ozone, diesel exhaust) by altering their cargo, including miRNAs, cytokines, and oxidative stress markers, mediating the effects of air pollutants (Alkoussa et al. 2020). Air pollutants tend to suppress EVs release, whereas heavy metals stimulate it. These environmental stressors enrich EVs with stress‐related proteins, cytokines, and microRNAs (miRNAs), altering their cargo profile. Moreover, EVs can capture and act as carriers of contaminants and allergens (Javdani‐Mallak and Salahshoori 2024; Long et al. 2025; Carberry and Rager 2023). These altered EV profiles can serve as early indicators of respiratory inflammation or oxidative damage, making them promising biosensors for air quality assessment.

In soil ecosystems, bacterial EVs (bEVs) mediate microbial communication and respond to pollutants such as heavy metals, pesticides and hydrocarbons. The presence and cargo of bEVs reflect environmental stress, allowing them to act as sensors of soil toxicity and microbial health (Ahmed and McKay; Yun et al. 2025). Marine organisms, including algae, invertebrates, and microbes, release EVs in response to contaminants like microplastics, oil spills and toxic algal blooms. These EVs carry molecular signatures (e.g., stress proteins, RNA species) that may be used to monitor ecosystem health and pollutant impact (Biller et al. 2023). EVs, especially bacterial membrane vesicles, can encapsulate and transfer ARGs between species across environments. Their detection in air, water or soil samples can serve as a sentinel system for monitoring the spread of antimicrobial resistance in contaminated areas (Yun et al. 2025).

Microplastics (MPs) have become omnipresent pollutants across marine, freshwater, terrestrial and atmospheric ecosystems, where they accumulate within living organisms. MPs have been shown to induce inflammation, oxidative stress and cellular toxicity, and may contribute to the development of diverse pathologies. In addition, they can act as vectors for chemical additives and environmental pollutants, further amplifying their potential health risks (Morrison et al. 2022; Landrigan et al. 2025). EVs function as carriers of MPs, mediating their intercellular transfer (Kim et al. 2024). Expelling MPs within EVs may be a defence strategy for donor cells to alleviate their toxicity, whereas simultaneous delivery to recipient cells can promote the distribution and accumulation of MPs within the surrounding microenvironment, ranging from neighbouring cells to the entire organism and, when widespread across species, ultimately impact whole ecosystems (Calzoni et al. 2024). Indeed, serum‐derived EVs content of immature pigs exposed to MPs suffered variations in the levels of metabolites involved in the regulation of lipid signalling pathways, disruption of glucose metabolism, mitochondrial dysfunction and impaired steroidogenesis (Mierzejewski et al. 2025). Detectable amounts of MPs in EVs and metabolomics changes after MPs exposition could serve as biomarkers for environmental pollution in higher organisms.

## 8 Energy Recovery and Remediation

Due to their abundance and versatility, EVs show strong potential for environmental applications, including bioremediation and waste treatment. However, further research is needed to fully understand their ecological roles and practical utility.

### 8.1 Soil and Water Remediation

Microorganisms and plants have been widely used in the bioremediation of heavy metals in soils. *Sporosarcina pasteurii*, a gram‐positive bacterium can tolerate silver stress to form nanoparticles and can remediate multiple heavy metals to promote the growth of various plants producing nanovesicles (PDNVs) that encapsulate nanosized particles in intra and extracellular spaces. This suggests that the bacteria evade the metal stress by converting the metal ions into nanosized particles and encapsulating them within nanovesicles to efflux them through the vesicle budding mechanism. Moreover, the bacteria increase secretion of EPSs to discharge the metal particles outside the bacterial system (Budamagunta et al. 2023).

Extracellular enzymes, mainly produced by microorganisms, play a crucial role in ecosystem processes by facilitating the degradation, transformation and mineralization of organic matter. Ecoenzymatic stoichiometry, the relative ratios of enzyme activities, is being increasingly explored as a novel approach to analyzing nutrient dynamics and serving as an indicator for monitoring and protecting ecosystem health (Luo et al. 2017). In this line, EVs secreted by microorganisms carry active enzymes that play a key role in nutrient detection and mobilization to make them available (Cai et al. 2023).

Together with microorganisms, plants are widely used in soil remediation (Mokrani et al. 2024) and their synergistic effects are also being explored (Masciandaro 2013). Plant‐microorganism interaction determines EVs production by the interacting species and, in turn, determine extracellular activity and pollutant degradation (Zhang et al. 2024).

Bioremediation of contaminated water bodies, such as rivers, lakes, wetlands and oceans relies on the natural capabilities of aquatic organisms, including microbes, algae, plants and invertebrates to degrade, detoxify or sequester pollutants. Aquatic microbes break down organic pollutants, certain bacteria can reduce toxic heavy metals, algae and cyanobacteria can take up nutrients (nitrogen, phosphorus), helping to prevent eutrophication, algae can adsorb heavy metals and other contaminants and provide oxygen to support aerobic bacterial degradation. Plants can absorb and accumulate heavy metals, nutrients, and organic pollutants and roots release exudates that stimulate microbial communities involved in pollutant degradation. Floating mats of macrophytes are used in engineered systems to treat contaminated water. Bivalves can filter and remove suspended contaminants, including microplastics and organic matter, activities of benthic invertebrates can aerate sediments, enhancing microbial degradation of pollutants (Ali 2023).

EVs from the organism used for water remediation could play an important role in the process of decontamination. EVs can carry active enzymes and redox‐active compounds that directly participate in pollutant degradation. They also carry signalling molecules for intercellular communication and synergy (Huang et al. 2023). It not irrational to think that EVs could increase degradation rates by extending enzyme activity beyond the cell, facilitate interactions by promoting communication among different microbial species, enhance stability through protection of biomolecules inside EVs and shuttle electrons, boosting system efficiency.

### 8.2 Biofilm Formation and Remediation

Biofilms are not only formed by microbial pathogens in host environments (Jiang et al. 2024), but also in controlled conditions with biotechnological uses (Ciofu et al. 2022). Biofilms are particularly applied in bioremediation of soil and water. The genetic arrangements within the biofilm lead to pollutant remediation, offering advantages such as high treatment efficiency, cost‐effectiveness and sustainability. Biofilms can develop in soil and water, and in polluted or ecosystems of extreme conditions, since community composition can adapt to the demand of the environment. Biofilms can be established in conditions with high concentrations in salts and xenobiotic contaminants, including heavy metals. Thus, they prove to be an excellent tool in polluted water bodies, groundwater bodies and organically polluted wastewater (Das et al. 2024).

As EVs of microbial pathogens are involved in biofilm development, they could also be a key player in adhesion, formation, organism interaction and symbiosis, dispersion and maturation of the architecture of biofilms in others milieu. This is a field of research to be explored.

### 8.3 E‐transfer

Electroactive microorganisms (EAMs) are ubiquitous in nature and have attracted considerable attention by their extracellular electron transfer (EET) capabilities. EAMs are those with the ability to exchange of electrons between intracellular and extracellular electron donors and acceptors (Zhao et al. 2021). EAMs are mainly used in synthesis of valuable chemicals, biotechnology, energy harvesting, biofilm formation and bioremediation as organic material degradation and wastewater treatment (Liu et al. 2014).

For EAMs, efficient EET is crucial for the sustainable and economically viable development of bioelectrochemical systems (BESs). However, low EET efficiency remains a significant bottleneck that limits the advancement of BESs. Additionally, the EET processes in many EAMs are not yet fully understood, leading to gaps in our knowledge of fundamental mechanisms. Key aspects that require further investigation include the mechanisms of electron transfer across microbial cell envelopes and the pathways of electron transfer between microbial surfaces and electrodes. Strategies to address these challenges include enhancing transmembrane electron transport, accelerating electron transport by promoting the synthesis and transmission of electron shuttles, and facilitating microbe‐electrode interfacial reactions by regulating biofilm formation.

EET mechanisms are found in bacteria, fungi, and archaea through redox molecules, although some EAMs also release OMVs to facilitate electron transfer to electrodes. OMVs that contain abundant cytochromes could promote electron exchange both between riboflavin and anodes and between cytochromes and anodes (Liu 2020). The role of such OMVs in energy transfer open new opportunities of environmental engineering.

## 9 Functional Food

EVs from natural sources (plants, probiotics, fermented foods) hold promise as bioactive agents in functional foods, offering opportunities for enhanced nutrition and wellness. They can be used as bioactive delivery vehicles and potential bioactive components themselves due to their relative stability within the human digestive tract given the lipid bilayer membrane that helps protect their cargo from harsh digestive conditions, including acidic gastric environments and digestive enzymes (Di Giulio et al. 2023). This structural resilience allows EVs to reach the intestines relatively intact, where they may interact with the gut epithelium or the microbiota. Understanding and optimizing this stability is crucial for harnessing EVs as delivery vehicles for bioactives and for maximizing their health‐promoting effects in functional foods and therapeutics (Turner 2024).

The nutritional value of food can be improved by the addition of bioactive compounds, such as polyphenols, vitamins and polyunsaturated fatty acids, which are often incorporated into foods to enhance their health benefits (Yeo 2023). However, the effectiveness of these bioactives is frequently compromised by poor bioavailability, which results from limited stability, solubility and structural transformations during digestion and absorption, as well as metabolic changes. One promising strategy to overcome these challenges is the incorporation of bioactives into nanoparticles, which can help protect these compounds and improve their delivery and absorption (Reiner and Somoza 2019). EVs from edible plants or PDNVs (e.g., fruits, vegetables, cereals) carry bioactive compounds like flavonoids, lipids, proteins, and sRNAs and exert health‐promoting anti‐inflammatory, antioxidant, anti‐cancer and regenerative properties (Di Gioia et al. 2020). Microbial EVs from probiotics (e.g., *Lactobacillus*, *Bifidobacterium*) in fermented foods (yogurt, kimchi, kefir) may contribute to gut health modulation and immune support (Krzyżek et al. 2023).

In recent years, agriwastes, such as fruit and vegetable peels, seeds and by‐products, have emerged as promising sources of edible EVs. By transforming low‐value waste into high‐value bioactive materials, this approach aligns with the principles of a circular economy. Furthermore, agriwaste‐derived EVs often contain bioactive compounds, including antioxidants and polyphenols, which can contribute to health benefits such as anti‐inflammatory and antioxidant effects. Compared to using fresh plant materials, leveraging agriwaste resources also reduces production costs, making this a sustainable and economically viable strategy (Latella et al. 2024).

Milk is a well‐recognized source of EVs, which carry bioactive cargos that can survive digestion and exert health‐promoting effects. Notably, these EVs can support immune system development and function, help maintain intestinal barrier integrity and modulate gut microbiota. Moreover, certain milk‐derived EVs exhibit anti‐inflammatory properties, offering potential benefits for inflammatory conditions, and they can also deliver regenerative signals that support tissue repair. The natural origin and biocompatibility of milk‐derived EVs make them attractive candidates for the development of functional foods and nutraceuticals. Their capacity to deliver bioactive cargos to target cells further expands their potential applications in promoting human health (Mecocci et al. 2022).

Standardizing isolation and characterization of food‐derived EVs, along with determining optimal dosages and validating their health benefits, is crucial for developing safe and effective functional food products. Regulatory frameworks, such as those from the ISEV, will be instrumental in ensuring product safety and guiding their integration into functional diets.

## 10 Large Scale Production and Standardization

Large‐scale production of EVs is a critical step for translating EV discoveries into practical applications. Massive production for applications, including therapeutics, cosmetics, EV‐based nanotechnology and nutraceutics, must consider controlling upstream and downstream technologies and process upscaling through the validation, standardization and regulatory issues along the process.

On this question, during the ‘massivEVs’ workshop, International Society for Extracellular Vesicles (ISEV, 2021), the scientific community on the field reached consensus on (1) prioritizing issues in large‐scale production in this order: function/potency/efficacy, safety and purity; and on (2) having a multi‐modal matrix of defined assays to establish QC of the EV product. In this workshop, the convenience of exploring potential non‐human EV sources such as bacteria, non‐human milk, microalgae and so forth, and interconnexion between different EV fields was discussed. Furthermore, collaboration between academia and industry was consider a met point mutually beneficial for all stakeholders (Paolini et al. 2022). On the other hand, ISEV Rigor and Standardization Subcommittee established a rigor and standardization framework that supports innovative EV research and applications, benefitting a wide range of stakeholders, from product developers to regulators (Welsh et al. 2024; Royo et al. 2020).

## 11 Future Perspective and Challenges

EVs are emerging as a rapidly advancing field, offering promising applications in medicine and veterinary science, dermatology and aesthetics, agriculture and environmental sustainability, as well as functional foods.

While the potential of EVs in drug delivery is widely recognized in human health (Hu et al. 2025), their use in veterinary medicine is delayed, although EVs hold also promise for disease diagnosis and therapy in livestock and companion animals (Moccia et al. 2022). Their role in host‐pathogen interactions could also provide insights into zoonotic disease transmission and control strategies (Li et al. 2024; Huang et al. 2023).

The potential of EVs in regenerative dermatology and aesthetic medicine is highly promising, given their ability to modulate skin regeneration, reduce scarring and combat ageing‐related changes (Novis et al. 2024). This paves the way for innovative, non‐invasive skincare and cosmetic treatments that will transform the cosmetic industry. When incorporated into cosmetic formulations they boost skin healing, hydration and protection while stimulating collagen production and reducing inflammation (Col et al. 2025). Furthermore, EVs can act as targeted delivery vehicles for active ingredients like antioxidants and peptides. Emerging research suggests that EVs based formulations could be personalized to individual genetics and skin needs and even used in minimally invasive treatments to speed up recovery (Costa and Santos 2017). Moreover, thanks to their ability to deliver bioactive molecules, EVs can also stimulate hair follicle cells, promote blood vessel growth (angiogenesis), reduce inflammation and activate stem cells to help hair regeneration. Ongoing research suggests EVs‐based treatments could become a key strategy to combat hair loss and enhance scalp health (Wu and Tang 2025). A source of EVs is attracting lot of attention and become even fashionable among wealthy people is the use of autolog plasma or EVs to assure compatibility and avoid rejection (Gupta et al. 2025). EVs derived from skin microbiota are being also explored for their cosmeceutical potential (Rajan et al. 2025).

The limitation in factorizing EVs from the logical and phylogenetically close‐relate sources and ethical issues has pressured significance in alternative sources. Mesenchymal stem cells (MSCs) are pivotal in regenerative medicine since this versatility makes them a powerful toolkit. MSCs can be isolated from multiple tissue types, including DPSCs, apical papilla stem cells (SCAPs), adipose‐derived stromal cells (ASCs), bone marrow MSCs (BMSCs), umbilical cord stem/stromal cells (UC‐SCs), and dermal‐derived stem cells (DSCs). EVs derived from these MSCs mimic many therapeutic effects of the parental MSCs (Davies et al. 2023; Zhu et al. 2025). Plant‐derived EVs or nanovesicles (PDNVs) have recently emerged as nanovehicles of actives for tissue repair and engineering due to the natural biocompatibility and low immunogenicity (Yuan et al. 2025). Thus, plants offer a sustainable and non‐toxic platform for producing EVs as nanovectors, while bacterial and fungal EVs show promise in vaccine development against infectious diseases (Moghaddam et al. 2025; Rodrigues et al. 2024). Hydrogel‐based microneedle patches containing EVs are emerging as a promising deep drug delivery system, with potential to improve healing in aged skin wounds and future clinical use (Wang et al. 2024).

PDNVs offer a wide range of novel approaches in the field and particularly for sustainable agriculture, offering natural tools for plant protection as delivering plant defence signals or modulating microbiome interactions, growth stimulation and resilience against biotic and abiotic stress, with potential to replace or reduce synthetic agrochemicals (Ambrosone et al. 2023). EVs could be involved in nutrient mobilization y competition, as fungi plant do, where enzymes imply in biogeochemical cycling are released to the extracellular media by EVs when needed, even stablishing interspecies interactions with other plants and microorganisms (Li et al. 2024). However, this is still an unexplored ground.

Edible EVs hold significant promise for future applications across health and wellness, driven by their natural origin, safety and ability to deliver bioactive cargo (Yang et al. 2023). In functional foods and nutraceuticals, edible EVs can be incorporated into next‐generation products that support gut health, immunity (Wu et al. 2024) and even mental well‐being through the gut‐brain axis (Sun et al. 2023; Yu et al. 2025). Emerging research suggests that these vesicles could be leveraged for precision nutrition, allowing for individualized dietary strategies based on genetic profiles, microbiome composition and specific metabolic needs. Furthermore, edible EVs offer a novel platform as natural drug delivery vehicles, capable of carrying small molecules, RNA therapeutics or peptides in a biocompatible, targeted manner (Langellotto et al. 2025). This opens new avenues for non‐invasive, holistic treatments that align with medicine approaches in human and animals.

Standardization and quality control remain critical challenges in advancing EV‐based therapies and other uses in humans. Despite the MISEV2023 guidelines (Welsh et al. 2024), harmonizing protocols for isolation, characterization and quantification across various settings remains difficult. The inherent variability and heterogeneity of EV populations further hinder reproducibility and clinical translation. Additionally, scalable manufacturing of clinical‐grade EVs with consistent purity and stability is essential to support therapeutic applications. Cargo loading and targeting are major technical challenges in EV‐based therapies. Effectively incorporating therapeutic agents while preserving EV structure and achieving precise delivery to target cells while avoiding unintended immune responses is essential for their safety and efficacy. Robust regulatory and safety frameworks are needed to guide the clinical translation of EV‐based therapies (Verma and Arora 2025). Thorough safety and immunogenicity assessments are crucial to ensure these therapies are safe, effective and consistent.

EVs from bacteria and fungi play roles in biofilm formation, environmental remediation and energy recovery. Understanding and leveraging these vesicles could support wastewater treatment, bioremediation and bioenergy production by modulating microbial communities or improving pollutant degradation (Ahmed and McKay 2024). On the other hand, EVs are advantageous as biosensors since non‐invasive sampling (e.g., from water or soil) is needed and present stability in harsh environmental conditions, cargo reflects specific stress responses and show potential for integration into bioelectronic sensors (Carberry and Rager 2023).

A growing interest in EVcology is coming (Huang et al. 2023; Biller 2020). EVs from both eukaryotic and prokaryotic organisms, where cell wall barriers add complexity, are implicated in key biological processes such as host‐pathogen interactions and dynamics, resistance transmission and plant pathogenesis. Advancing our understanding in EV biogenesis and inter‐species and interkingdom EV communication can open new avenues in EV potential exploitation as pest control, disease prevention, therapeutic innovation and, definitively, EV safe production.

This article explores active and emerging EV applications, outlining the promising avenues for future development in the sections that follow. Overall, while the potential of EV‐based applications across regenerative medicine, skincare and immunomodulation is undeniable, progress depends on addressing critical challenges in standardization, regulatory oversight, mechanistic understanding and delivery targeting. As the field matures, a multidisciplinary approach, bridging cell biology, immunology, nanotechnology and clinical research, as well as engineering, ecology understanding will be essential for unlocking the full therapeutic promise of EVs.

## Author Contributions

**Juan Manuel Falcón‐Pérez**: conceive the study, supervision, critical revisions, review and editing and funding acquisition. **Esperanza González**: design the review structure, performed the literature search, drafted the manuscript, visualization and project administration. All authors read and approved the final version of the manuscript.

## Funding

This work was supported by The European Union (EVCA: 101079264) and MICIU/AEI /10.13039/501100011033 and FEDER, UE (Plan Nacional: PID2024‐155622OB‐I00).

## Conflicts of Interest

The authors declare no conflicts of interest.

## Data Availability

Data sharing not applicable to this article as no datasets were generated or analysed during the current study.
